# Evaluation of a Biopsy Gun for Guided Biopsy of Impalpable Liver Lesions Using Intraoperative Utrasound

**DOI:** 10.1155/1990/56823

**Published:** 1990

**Authors:** R. M. Charnley, J. P. Sheffield, J. D. Hardcastle

**Affiliations:** 1 Department of Surgery University Hospital Nottingham NG7 2UH United Kingdom; 2 Department of Pathology University Hospital Nottingham NG7 2UH United Kingdom; 3 Department of Surgery E Floor, West Block University Hospital Nottingham NG7 2UH United Kingdom

## Abstract

A biopsy gun which can be operated by one hand has been evaluated at post-mortem to determine its accuracy in biopsying impalpable lesions within the liver under intraoperative ultrasound control. Of 20 impalpable metastases identified positive histology was obtained in 90% demonstrating that this technique is of value in identifying and localising metastases in the liver.


HPB Surgery, 1990, Vol. 2, pp. 265–267
Reprints available directly from the publisher
Photocopying permitted by license only

© 1990 Harwood Academic Publishers GmbH
Printed in the United Kingdom

### EVALUATION OF A BIOPSY GUN FOR GUIDED BIOPSY OF IMPALPABLE LIVER LESIONS USING INTRAOPERATIVE UTRASOUND

R.M. CHARNLEY, *J.P. SHEFFIELD, J.D. HARDCASTLE

*Departments of Surgery and *Pathology, University Hospital, Nottingham NG7 2UH.*

*(Received 16 November 1989)*

A biopsy gun which can be operated by one hand has been evaluated at post-mortem to determine its accuracy in biopsying impalpable lesions within the liver under intraoperative ultrasound control. Of 20 impalpable metastases identified positive histology was obtained in 90% demonstrating that this technique is of value in identifying and localising metastases in the liver.

KEYWORDS: Occult hepatic metastases, intraoperative ultrasound, ultrasound guided biopsy

### INTRODUCTION

As many as 35% of patients who undergo curative resection for colorectal cancer have occult liver metastases which are undetected at operation [1,2]. Intraoperative ultrasound has the ability to detect a certain proportion of these occult metastases at the time of surgery [3] and this may improve the possibility of cure by hepatic resection. Identification of impalpable metastases within the liver relies not only on imaging the lesions but also on gaining histological evidence by biopsy.

We have evaluated a biopsy gun for guided biopsy of impalpable liver metastases by assessing the accuracy of guided biopsy at post-mortem.

### METHOD

Impalpable liver metastases were identified at post-mortem by contact ultrasound using an intraoperative ultrasound probe containing a 7mHz transducer (Bruel and Kjaer 1846 and 8537) with a focus at 1 – 6 cm and a high resolution capable of detecting metastases at operation as small as 5 mm. Many impalpable metastases were identified. Twenty metastases were individually measured using the light-pen attachment on the ultrasound machine and each lesion was biopsied under ultrasound control using a biopsy gun (Biopty-Radiplast AB, Uppsala, Sweden) which can be held in one hand while the other hand is free to hold the intraoperative ultrasound probe. The biopsy needle (18-gauge) was inserted parallel to the

Correspondence to: R. M. Charnley, Department of Surgery, E Floor, West Block, University Hospital, Nottingham, NG7 2UH.

axis of the ultrasound probe and was easily positioned (Figure 1) without the use of a special biopsy attachment. The gun has a rapid firing action and this ensures that a relatively atraumatic biopsy is taken. The 20 core biopsies obtained using this technique were individually analysed histologically for the presence of carcinoma.

### RESULTS

The 20 impalpable metastases identified measured between 0.9 cm and 1.8 cm in diameter with a median diameter of 1.2 cm. The lesions were between 2 cm and 5 cm from the liver surface. Histology of the biopsies obtained revealed the presence of carcinoma in 18/20 biopsies. The 2 metastases which were missed by the biopsy needle were 0.9 cm and 1.1 cm in diameter.

### DISCUSSION

The use of intraoperative hepatic ultrasound during surgery for colorectal cancer is becoming widely accepted as an accurate technique for the detection of impalpable liver metastases. The combination of intraoperative ultrasound and guided biopsy of the liver enables the surgeon to accurately stage each patient and obtain

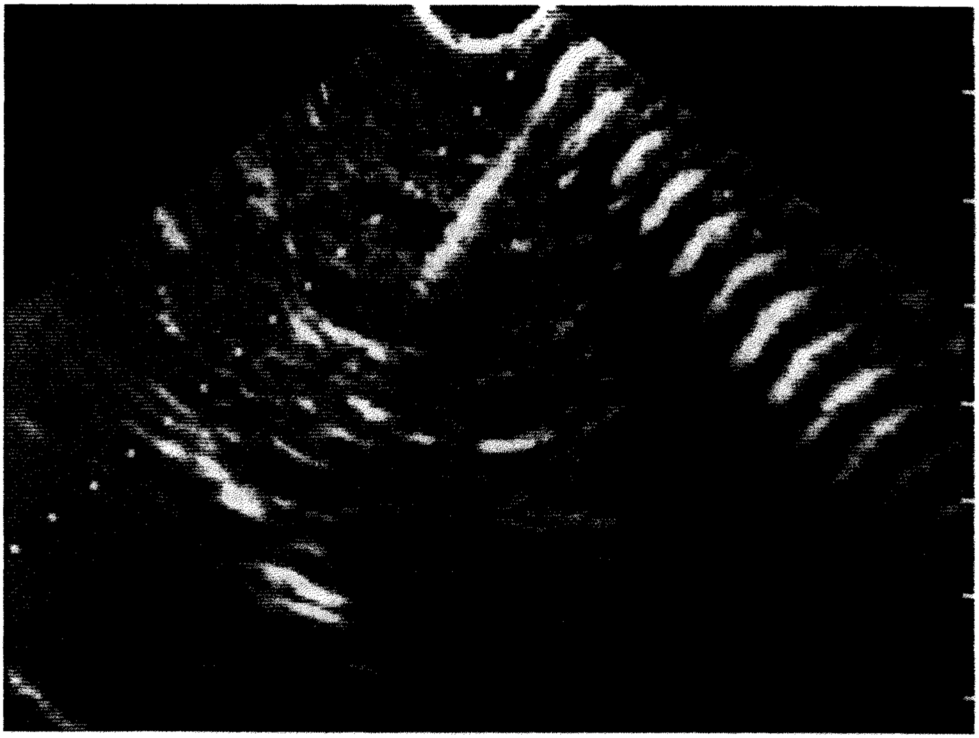

**Figure 1** Biopsy gun needle guided into position under intraoperative ultrasound control.

histological proof of the majority of the lesions detected. Contact ultrasound of the post mortem liver has previously been shown to detect 100% of metastases greater than 1.5 cm in diameter, 85% of metastases 1.0 – 1.4 cm in diameter and 66% of metastases 0.5 – 0.9 cm in diameter [5]. We have now demonstrated that accurate localisation and biopsy of impalpable metastases down to a diameter of 0.9 cm is possible using a biopsy gun under ultrasound control. The particular advantage of this type of gun over a standard biopsy needle is that it takes a relatively atraumatic biopsy and only one hand is required to insert the needle and take the biopsy leaving the other hand free to hold the intraoperative ultrasound probe and localise the lesion for biopsy. With adequate exposure at operation a lesion in any part of the liver may be biopsied.

The use of a biopsy gun is to be recommended for the guided biopsy of impalpable lesions under intraoperative ultrasound control.

*References*

1. Finlay, I.G., McArdle, C.S. (1986) Occult hepatic metastases in colorectal carcinoma. *Br J Surg* **73**, 732–735.
2. Leveson, S.H., Wiggins, P.A., Giles, G.R., Parkin, A., Robinson, P.J. (1985) Deranged liver blood flow patterns in the detection of liver metastases. *Br J Surg* **72**, 128–130.
3. Boldrini, G., de Gaetano, A.M., Giovannini, I., Castagneto, M., Colagrande, C., Castiglioni, G. (1987) The systematic use of operative ultrasound for detection of liver metastases during colorectal surgery. *World J Surg* **11**, 622–627.
4. Machin, J., Isomoto, H., Yamashita, Y., Kurohiji, T., Shirouzo, K., Kakegawa, T. (1987) Intraoperative ultrasonography in screening for liver metastases from colorectal cancer: comparative accuracy with traditional procedures. *Surgery* **101**, 678–684.
5. Thomas, W.M., Morris, D.L., Hardcastle, J.D. (1987) Contact ultrasonography in the detection of liver metastases from colorectal cancer: an *in vitro* study. *Br J Surg* **74**, 955–956.

### INVITED COMMENTARY

It has generally been said that surgeons are slow to adopt new technology and that we have diagnosed and treated surgical diseases in the same fashion for decades. This is certainly not true any more when it comes to the treatment of liver tumours. The widespread use of the ultrasound knife in liver resection has made this operation much safer and more widely applicable. With the advent of intraoperative ultrasound the number of patients that could be treated and cured will probably be markedly increased. If the impressive data shown in this "experimental" study is also found to be true in clinical practice then it will be possible to diagnose very small synchronous liver metastases. It is hoped that this will increase the long-term survival of patients with colorectal metastatic disease. The authors of the article are to be congratulated for an excellent approach to this problem and we are looking forward to further clinical studies.

Bengt Jeppsson, M.D., Ph.D.
Associate Professor of Surgery
Department of Surgery, Lund University, S-221 85 LUND, Sweden

